# Development of an Immunochromatographic Strip for Rapid Detection of *Pantoea stewartii subsp. stewartii*

**DOI:** 10.3390/s150204291

**Published:** 2015-02-12

**Authors:** Min Feng, Dezhao Kong, Wenbing Wang, Liqiang Liu, Shanshan Song, Chuanlai Xu

**Affiliations:** 1 Huaian Entry-Exit Inspection and Quarantine Bureau, Huaian 223001, China; E-Mail: fm_8228@163.com; 2 State Key Lab of Food Science and Technology, School of Food Science and Technology, Jiangnan University, Wuxi 214122, China; E-Mails: kdz19900910@163.com (D.K.); wenbin66@yeah.net (W.W.); raxray@gmail.com (L.L.); songshanshan0626@126.com (S.S.)

**Keywords:** *Pantoea stewartii subsp. stewartii* (Pss), immunochromatographic test strip, monoclonal antibody

## Abstract

A rapid, simple, sensitive, and specific immunochromatographic test strip was developed for the detection of *Pantoea stewartii subsp. stewartii* (Pss) in corn seed which was soaked overnight and then centrifuged for precipitate re-dissolved as samples. A pair of sensitive monoclonal antibodies for the immunochromatographic test strip was generated by mice immunization and cell fusion. Under optimized conditions, the lower detection limit of the strips for Pss was 1 × 10^5^ cfu/mL both in 0.01 M phosphate buffer solution and corn seed samples, with no cross-reactivity with other common plant pathogens. The developed strip is useful and rapid for the detection of Pss in corn seed samples.

## 1. Introduction

Stewart's bacterial wilt of corn is a devastating plant disease caused by *Pantoea stewartii subsp. stewartii* (Pss), a Gram-negative facultative anaerobic bacillus of the genus *Pantoea* [1]. The disease was first detected in sweet corn by Stewart in 1895 in New York, USA and eventually spread to other parts of the world [2,3]. The pathogenic bacterium infects corn at each vegetative stage and spreads mainly through the corn flea beetle. However, it is also present in internal and external seed sections [4,5]; therefore, contaminated seeds represent the main transmission route of the plant pathogen on the international trade. The importation of corn seeds has been banned in several countries unless the seeds are certified Pss-free.

Conventional field observation and biochemical detection methods are not sensitive enough to detect the presence of Pss in seeds because of the invisible characteristic symptoms. Additionally, these detection methods are repetition and trade and the labor-intensive and time-consuming [6]. Molecular biological techniques and immunological methods have been used for the detection of plant pathogens: polymerase chain reaction [7,8], loop-mediated isothermal amplification [9], immunosensor analyses [10–12], and enzyme-linked immunosorbent assay (ELISA) [13]. DNA-based detection methods are highly sensitive but require several extraction steps, specific instruments, and trained operators. ELISA is a simple, specific, and low-cost method commonly used for pathogen detection [14]; however, it is time-consuming.

Lateral-flow immunochromatographic strip assays are rapid, simple, inexpensive, and instrument-free diagnostic tools. Following a 5–10 min reaction, the results can be obtained with the naked eye [15–17]. Colloidal gold nanoparticles are ideal biological tags for bio-recognition because of their ease in conjugation reactions [18]. Additionally, lanthanide chelates can be used through fluorogenic reactions [19].

In China, Pss have not been detected. However, there is a high risk for Pss-contaminated seeds imported into China. Therefore, the development of a rapid and accurate detection method is important. In this study, antibodies were obtained following mice immunization and cell fusion and used in an immunochromatographic lateral-flow strip for the detection of Pss.

## 2. Material and Methods

### 2.1. Bacterial Strains and Chemicals

The bacterial strains used in this study (*Pantoea stewartii subsp. stewartii* NCPPB 449, Burkholderia glumae NCPPB 3591, Xanthomonas oryzae pv. oryzicola NCPPB 1150, Pseudomonas syringae pv. syringae NCPPB 2844, and Xanthomonas oryzae pv. oryzae NCPPB 3002) were obtained from the Hunan Entry-Exit Inspection and Quarantine Bureau (Changsha, China). Complete Freund's adjuvant, incomplete Freund's adjuvant, and enzyme immunoassay-grade horseradish peroxidase-labeled goat anti-mouse immunoglobulin were obtained from Sigma (St. Louis, MO, USA). Gelatin was purchased from Beijing Biodee Biotechnology Co., Ltd. (Beijing, China). Tetramethylbenzidine and horseradish peroxidase (HRP) were purchased from Aladdin Chemistry Co., Ltd. (Shanghai, China). All reagents for cell fusion were obtained from Sunshine Biotechnology Co., Ltd. (Nanjing, China). Nutrient broth yeast medium (NBY) was obtained from Beijing Land Bridge Technology Co., Ltd. (Beijing, China). Other reagents and chemicals were obtained from the National Pharmaceutical Group Chemical Reagent Co., Ltd. (Shanghai, China). The nitrocellulose high-flow plus membrane (Pura-bind RP) was obtained from Whatman-Xinhua Filter Paper Co., Ltd. (Hangzhou, China). The glass fiber membrane (CB-SB08), the polyvinylchloride backing material, and the absorbance pad (SX18) were supplied by Goldbio Tech Co., Ltd. (Shanghai, China).

### 2.2. Preparation of Monoclonal Antibody (mAb) against Pss

#### 2.2.1. Pantoea stewartii subsp. stewartii (Pss)

Pss NCPPB 449 was selected as the immunogen. The cryopreserved strain was activated in lysogeny broth medium (pH 7.0) at 28 °C for 2 d and inoculated on nutrient agar plate at 28 °C for 2 d. Inoculation was performed with one colony in NBY medium (pH 7.0) at 28 °C for 2 d.

#### 2.2.2. Immunization and mAb

Five female BALB/c mice (6 weeks old) were immunized subcutaneously with 150 μL of 10^8^ cfu/mL heat-destroyed Pss mixed with an equal volume of Freund's complete adjuvant (Freund's incomplete adjuvant was used in subsequent immunizations). Immunization was repeated every three weeks until a high serum antibody titer was obtained based on indirect ELISA results [14]. The mouse with the highest serum titer was sacrificed, and mouse spleen cells were fused with SP2/0 myeloma cells. Positive hybridoma cell lines were determined via indirect ELISA screening after sub-cloning. Positive hybridoma cells were injected into BALB/c mice for mAb production [20]. Antibodies were purified from ascites by caprylic acid-ammonium sulfate precipitation and conjugated to HRP by the sodium periodate method [21]. The mAb combinations were assessed by sandwich ELISA and used as capture antibody and gold-labeled antibody, respectively, in the immunochromatographic strip.

### 2.3. Development of the Immunochromatographic Strip

#### 2.3.1. Colloidal Gold Nanoparticle

**Figure 1.**
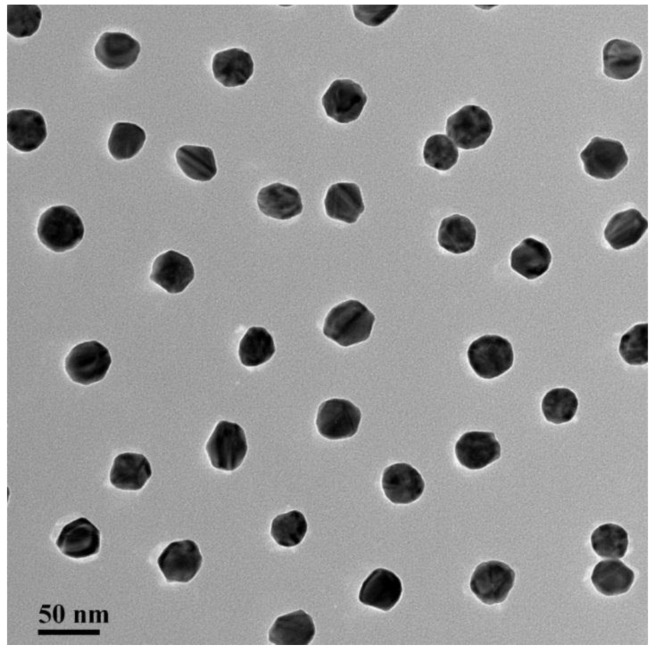
The TEM of colloidal gold nanoparticle (30 nm).

Colloidal gold particles were prepared [22]. Briefly, 200 mL of 0.1 g/L chlorauric acid was heated to boiling under constant stirring (100 g), mixed with 8.0 mL of 1% trisodium citrate (w/v) at 300 °C, and stirred for 10 min until the color of the solution turned from yellow to wine-red. The solution was allowed to cool at room temperature under constant stirring and stored at 4 °C. All solvents were prepared with deionized water. Transmission electron microscopy (TEM) examinations revealed that the gold nanoparticles had uniform particle size (approximately 30 nm in diameter; Figure 1).

#### 2.3.2. Gold Nanoparticle-Labeled mAb

Through the negative charge on surface, Colloidal gold particles can quickly and steadily adsorbed the positively charged polymer material such as proteins and does not destroy its biological activity. The ion concentration and the ratio of both material would impacted the adsorption of colloidal gold to proteins [23,24]. The colloidal gold solution was adjusted to pH 7.0 with 0.1 M K_2_CO_3_. Subsequently, 0.16 mg mAb in phosphate buffer solution (PBS) at pH 7.4 was added dropwise into 10 mL colloidal gold nanoparticle solution and kept at room temperature for 50 min. One milliliter of 0.5% BSA (w/v) was slowly added into the solution to block the gold nanoparticles and stabilize the labeled mAb. Following a 2 h incubation, the solution was centrifuged at 7000 g for 30 min. The resulting precipitate was washed three times with 0.02 M PBS (containing 5% sucrose, 1% BSA, and 0.5% PEG 6000, pH 7.4), dissolved in 5 mL of 0.02 M PBS (containing 0.02% NaN_3_), and stored at 4 °C [22].

#### 2.3.3. Immunochromatographic Strip Preparation

**Figure 2.**
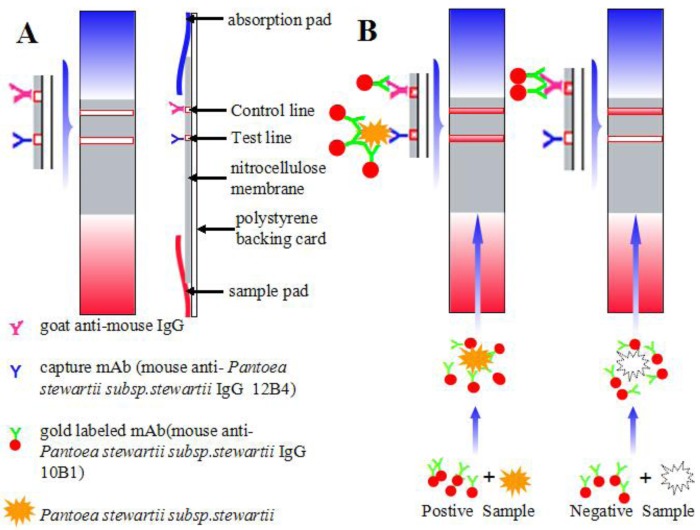
The schematic image of the assembled strip and the principle of the detection. (**A**) The schematic image of the assembled strip; (**B**) The principle of the detection method.

The immunochromatographic strip consisted of four parts: the sample pad, the nitrocellulose membrane, the polystyrene backing card, and the absorption pad, which were assembled in layers (Figure 2A). The capture mAb was used in the test line (T line) to detect the presence of pathogens in the samples; goat anti-mouse IgG was used in the control line (C line). These immunoglobulins were sprayed onto the nitrocellulose (NC) membrane at 1 μL/cm using a membrane dispenser (XinqidianGene-Technology Co. Ltd., Beijing, China) at the concentration of 4 mg/mL to capture mAb and 0.5 mg/mL to the goat anti-mouse IgG, respectively [25]. The NC membrane was dried at 37 °C for 30 min. The sample pad was immersed in PBS (containing 1% BSA and 0.2% Tween 20) and dried at 37 °C for 4 h to minimize nonspecific binding and matrix interference. The assembled strips were placed into a plastic drum prior to use.

#### 2.3.4. Immunochromatographic Assay

This immunochromatographic assay was based on an antibody pair: one type of mAb was conjugated to gold nanoparticle (GNP) as the detection antibody and the other type of mAb was sprayed onto the NC membrane as the capture antibody. Prior to the test, GNP-mAb was added to the sample and allowed to react at room temperature for 5 min. The reaction solution was added to the sample pad of the strip. After 5 min, the results were observed with the naked eye. If the sample contains pathogens, GNP-mAb binds to the pathogens. Through capillary action, the reaction solution flows to the absorption pad, where the capture antibody immobilized at the T line captures the GNP-mAb-pathogens. With the deposition of GNP-mAb-pathogens, a red line appears on the NC membrane.

A sample is positive for Pss if two lines appear: T and C. A sample is negative for Pss if only the C line appears (Figure 2B). The intensity of the T line reflects the amount of captured GNP-mAb-pathogens. The more pathogens are captured, the greater the GNP-mAb interaction and the higher the color intensity on the T line. The C line should always appear; otherwise, the procedure was incorrectly carried out or the strip was invalid and a repeat test with a new strip should be performed [26].

### 2.4. Sample Analyses

#### 2.4.1. Sensitivity and Specificity

The sensitivity of the immunochromatographic strip was determined by standard testing. Heat-destroyed Pss NCPPB 449 were diluted to 2 × 10^7^, 3.3 × 10^6^, 1 × 10^6^, 3.3 × 10^5^ and 1 × 10^5^ cfu/mL in 0.01 M PBS (pH 7.4). The limit of detection was determined. A series of common plant pathogens were diluted to different concentrations (1 × 10^8^, 1 × 10^7^, 1 × 10^6^ and 1 ×1 0^5^ cfu/mL) and subjected to the immunochromatographic strip. The blank sample consisted of 0.01 M PBS. In this experiment, 50 μL GNP-mAb was mixed with 150 μL sample, allowed to react at room temperature for 5 min, and added to the sample pad of the strip. Following a 5-min incubation, the T and C lines were observed with the naked eye. The lower detection limit (LDL) was defined as the concentration of bacteria that was clearly visible on the T line. Tests were repeated six times at each concentration [27].

#### 2.4.2. Detection of Pss-Spiked Corn Seeds

In this experiment, 500 g of corn seeds was immersed in 0.5% NaCl for 5–15 min, washed three times, and dried under aseptic conditions. The seeds were smashed following surface disinfection and immersed overnight in 0.9% NaCl at 4 °C. The solution was centrifuged at 4000 g for 10 min; the resulting supernatant was centrifuged at 10,000 g for 15 min. The precipitate was re-dissolved in 2 mL 0.9% NaCl. This suspension was negative for Pss (confirmed by immune separation methods against the reference standard SN/T 1375-2004), and spiked with 1 × 10^7^, 1 × 10^6^, or 1 × 10^5^ cfu/mL Pss. Each concentration was repeated three times.

## 3. Results and Discussion

### 3.1. mAbs for the Immunochromatographic Strip

The mAbs for the immunochromatographic strip were selected by the sandwich ELISA method. The optimal antibody combination was selected by pair-wise interaction analysis (Supplementary Figure S1). The antibody 12B4 was used as the capture antibody; the antibody 10B1 was used as the detection antibody. By this combination, the limit of detection was 1.5 × 10^4^ cfu/mL Pss, with a linear range of 4.57 × 10^5^ to 1.11 × 10^8^ cfu/mL (Supplementary Figure S2) and has no cross-reactivity with any of the four bacterial strains (Supplementary Table S1).

### 3.2. Characterization and Optimization of the Immunochromatographic Strip

GNP was selected as the detection signal in the immunochromatographic strip. The GNP-labeled antibody deposited on the surface of the NC membrane and formed a visible red line without any substrates. The intensity of the color was proportional to the concentration of the standard.

**Figure 3.**
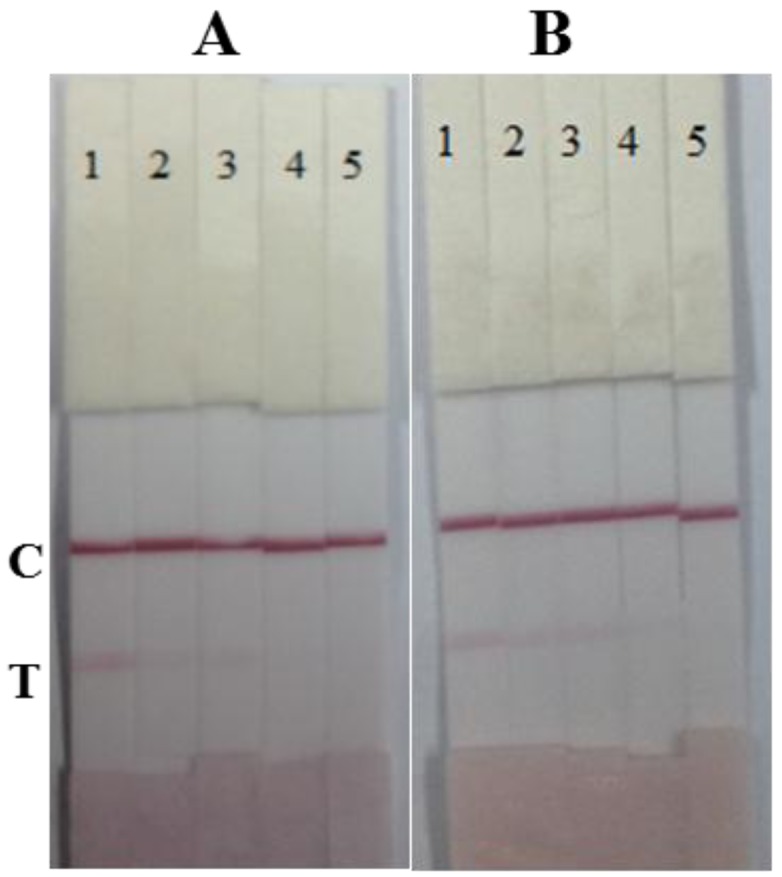
The optimization of antibodies for immunochromatographic strip. (**A**) Antibody 10B1 as the capture antibody and 12B4 as the gold-labeled antibody; (**B**) Antibody 12B4 as the capture antibody and 10B1 as the gold-labeled antibody; the concentration was as follows: (1) 1 × 10^7^, (2) 3.3 × 10^6^, (3) 1 × 10^6^, (4) 3.3 × 10^5^ cfu/mL and (5) blank. T, test line. C, control line.

The antibodies 12B4 and 10B1 were both used as capture antibodies, and tested with a series of bacterial standards (1 × 10^7^, 3.3 × 10^6^, 1 × 10^6^, and 3.3 × 10^5^ cfu/mL in 0.01 M PBS). The antibody 12B4 was chosen as the capture antibody and the antibody 10B1 was chosen as the gold-labeled antibody because of the lower LDL of the strip (Figure 3).

Additionally, gold nanoparticles with a diameter of 30 nm were selected because of their stability, simplicity, and bright color [28,29]. One milliliter of gold nanoparticle solution with 4 μL 0.1 M K_2_CO_3_ was used to stabilize the colloidal gold solution and antibody 10B1. The capture antibody 12B4 had a concentration of 4 mg/mL on the T line, and the goat anti-mouse IgG had a concentration of 0.5 mg/mL on the C line. The block buffer for the sample pad was 0.01 M PBS containing 1% BSA and 0.2% Tween 20 to prevent nonspecific binding and matrix interference [30].

**Figure 4.**
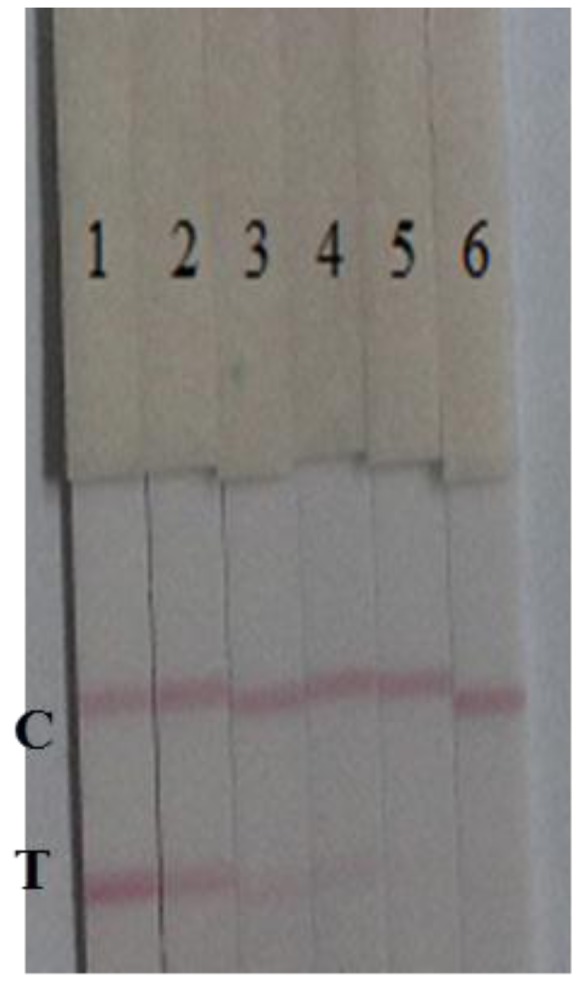
The detection of Pss by immunochromatographic strip. The concentration was as follows: (1) 1 × 10^7^, (2) 3.3 × 10^6^, (3) 1 × 10^6^, (4) 3.3 × 10^5^, (5) 1 × 10^5^ cfu/mL and (6) blank. T, test line. C, control line.

The assay sensitivity was confirmed with a series of diluted standards. GNP-mAb (50 μL) was mixed with sample solution (150 μL), allowed to react at room temperature for 5 min, and added to the sample pad of the strip. The concentrations of the standards were 1 × 10^7^, 3.3 × 10^6^, 1 × 10^6^, 3.3 × 10^5^, and 1 × 10^5^ cfu/mL Pss in 0.01 M PBS (pH 7.4). The results are shown in Figure 4.

The LDL was set at the Pss concentration that resulted in a visible red color T line. After a 5-min reaction, a visible red T line was obtained with 1 × 10^5^ cfu/mL Pss; the color intensity of the T line was proportional to Pss concentrations.

#### 3.3. Specificity of the Strip Test

**Figure 5.**
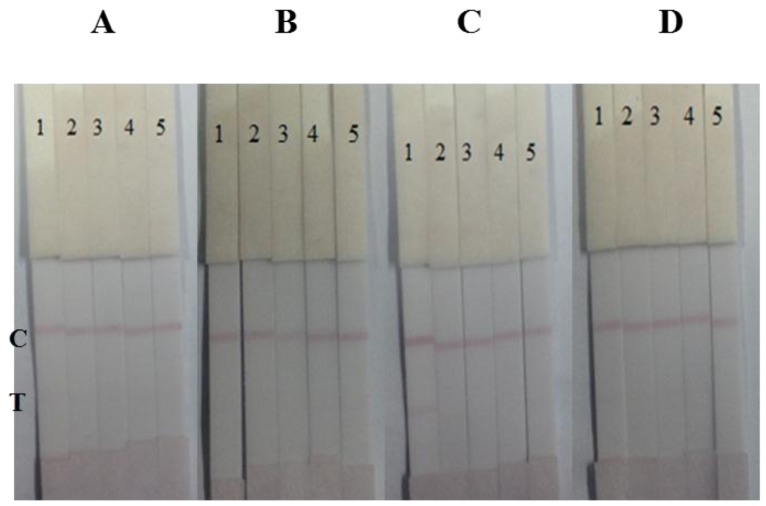
The cross-reaction of the immunochromatographic strip (**A**) *Burkholderia glumae* NCPPB 3591; (**B**) *Xanthomonas oryzae pv. Oryzicola* NCPPB 1150; (**C**) *Pseudomonas syringae pv.syringae* NCPPB 2844; (**D**) *Xanthomonas Oryzae pv.Oryzae* NCPPB 3002; the concentration was as follows: (1) 1 × 10^8^, (2) 1 × 10^7^, (3) 1 × 10^6^, (4) 1 × 10^5^ cfu/mL and (5) blank. T, test line. C, control line.

The specificity of the immunochromatographic strip was determined in the presence of four plant pathogens: *B. glumae* NCPPB 3591, *X. oryzae pv. oryzicola* NCPPB 1150, *P. syringae pv. syringae* NCPPB 2844, and *X. oryzae pv. oryzae* NCPPB 3002, at different concentrations (1 × 10^8^, 1 × 10^7^, 1 × 10^6^ and 1 × 10^5^ cfu/mL in 0.01 M PBS, pH 7.4). The results are shown in Figure 5.

The results revealed that there was no cross-reactivity with any of the four bacterial strains. At 1 × 10^8^ cfu/mL *Oryzicola* NCPPB 1150, a weak color was observed. However, the cross-reactivity of the immunochromatographic strip with *Oryzicola* was 0.1% (cross-reactivity = LDL of Pss/LDL of *Oryzicola* NCPPB 1150). Therefore, the developed immunochromatograhic strip can be used for Pss detection due to its specificity, speed, simplicity, and low-cost.

### 3.4. Detection of Pss-Spiked Corn Seed Samples

**Figure 6.**
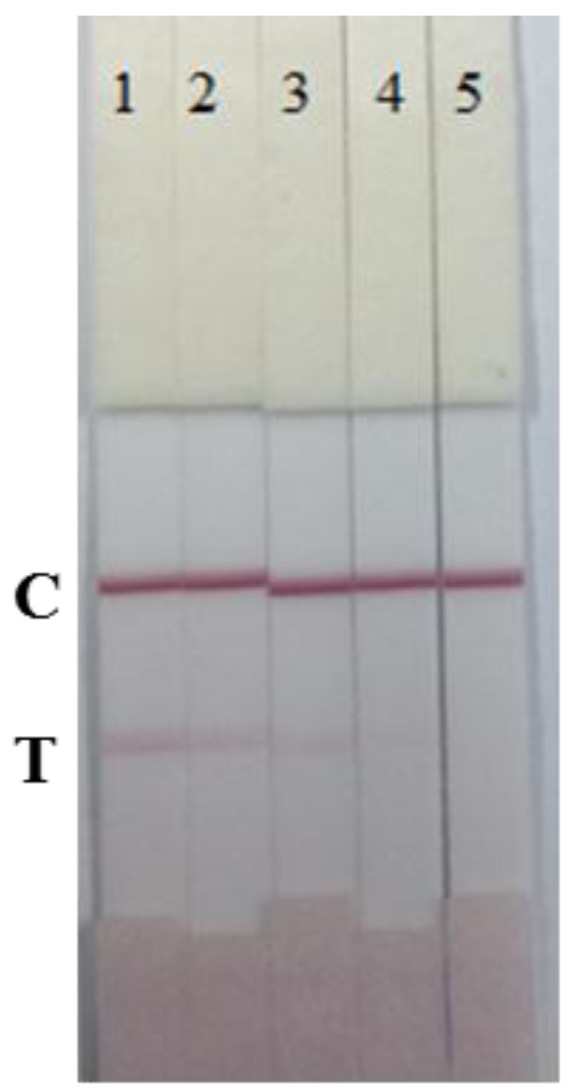
Detection of Pss-spiked corn seed samples by immunochromatographic strip. The concentration was as follows: (1) 1 × 10^7^, (2) 3.3 × 10^6^, (3) 1 × 10^6^, (4) 1 × 10^5^ cfu/mL and (5) blank. T, test line. C, control line.

Pss-free corn seed samples were spiked with Pss (final concentrations: 1 × 10^7^, 3.3 × 10^6^, 1 × 10^6^, and 1 × 10^5^ cfu/mL). The LDL of the strips was 1 × 10^5^ cfu/mL. However, due to matrix effects, the strip containing corn seeds showed a T line weaker than that with 0.01 M PBS (Figure 6).

Compared with the conventional sandwich ELISA method used the same mAbs in our work (with a linear range of 4.57 × 10^5^ to 1.11 × 10^8^ cfu/mL), the colloidal gold-based strip was more rapid and has a more sensitive result. The fluorescence strip developed by Zhang Fan at 2014 [19] based on lanthanide chelates to Pss was capable of detecting a minimum of 10^3^ cfu/mL in 20 min. However, the result was obtained by the test strip reader devised by their group and with the complex Synthesis process of lanthanide-coated nanoparticles. The microsphere immunoreaction method developed by Zhang Fan at 2013 [11] has the qualitative detections of Pss 10 times lower than ELISA. With a shorter analysis time (1.5 h). By the compare, the colloidal gold-based strip was benefits in the low fabrication costs, user-friendly format, rapid detection process, small sample need, free equipment requirement.

## 4. Conclusions

A sensitive and specific colloidal gold-based lateral-flow immunochromatography strip was developed for the rapid detection of Pss in corn seed samples. A pair of highly sensitive and specific anti-Pss antibodies was obtained from mice immunization and cell fusion. The results revealed that the LDL of Pss was 1 × 10^5^ cfu/mL both in 0.01 M PBS and corn seeds. There was no cross-reactivity with any of the four bacterial strains tested. However, the intensity of the T line was weaker in corn seed samples compared to that in 0.01 M PBS because of matrix effects. The colloidal gold-based immunochromatographic strip is a simple, rapid, and sensitive method for the detection of Pss in corn seeds. The whole process was completed within 10 min. This method could have potential applications in the evaluation of imported corn seeds.

## Supplementary Material
