# Supplementary material for: Development of an Immunochromatographic Strip for Rapid Detection of Pantoea stewartii subsp. stewartii
Source: Sensors (Basel). 2015 Feb 12;15(2):4291–301. doi: 10.3390/s150204291 (PMC4367412; doi:10.3390/s150204291)
Supplement: Supplementary file 1 [file sensors-15-04291-s001.pdf]

OPEN ACCESS
sensors
ISSN 1424-8220
www.mdpi.com/journal/sensors

Supplementary Information

Development of an Immunochromatographic Strip for Rapid Detection of *Pantoea stewartii subsp.stewartii*. *Sensors* 2015, *15*, 4291-4301

Min Feng [1,2], Dezhao Kong [2], Wenbing Wang [2], Liqiang Liu [2], Shanshan Song [2] and Chuanlai Xu [2,*]

[1] Huaian Entry-Exit Inspection and Quarantine Bureau, Huaian 223001, China;
E-Mail: fm_8228@163.com

[2] State Key Lab of Food Science and Technology, School of Food Science and Technology, Jiangnan University, Wuxi 214122, China; E-Mails: kdz19900910@163.com (D.K.); wenbin66@yeah.net (W.W.); raxray@gmail.com (L.L.); songshanshan0626@126.com (S.S.)

* Author to whom correspondence should be addressed; E-Mail: xcl@jiangnan.edu.cn;
Tel.: +86-510-8532-9076.

1. Experimental Section

1.1. Buffers and Solutions

0.05 M sodium carbonate-bicarbonate buffer (CBS, pH 9.6); 0.05 M sodium carbonate-bicarbonate buffer containing 0.2% (w/v) gelatin as blocking buffer; 0.01 M phosphate buffered saline (PBS, pH 7.4); 0.01 M phosphate buffered saline containing 0.05% (v/v) Tween-20 (PBST, pH 7.4); 0.01 M phosphate-buffered saline containing 0.1% (w/v) gelatin as antibody dilution; 0.1 M citrate phosphate buffer (pH 5.0) containing 180 µL of 30% H_2O_2 (A solution) and ethylene glycol substrate solution containing 0.06% (w/v) 3,3',5,5'-tetramethylbenzidine (B solution), mixed at a ratio of 5:1 as substrate solution; 2 M sulfuric acid as stop reagent.

1.2. Development of Monoclonal Sandwich ELISA Method

The procedure of sandwich ELISA method was as follows: microtiter plates were coated with capture mAb at 37 °C for 2 h with 100 µL/well in CBS (pH 9.6). Plates were washed three times with PBST after incubation and then incubated with blocking buffer at 37 °C for 2 h (200 µL/well). After washing three times, plates were incubated with heat-killed *Pantoea stewartii subsp.stewartii* in 0.01 M PBS or blank (0.01 M PBS) at 37 °C for 1 h (100 µL/well). Then the plates were washed three times and incubated with HRP-mAb in antibody dilution at 37 °C for 1 h (100 µL/well). After washing four times, 100 µL/well substrate solution was added and plates were incubated at 37 °C for 15 min in dark and then stopped by 50 µL/well stop reagent. The absorbance at 450 nm was determined by a microtiter plate reader (BioTek, Winooski, VT, USA).

1.3. Pair-Wise Interaction Analysis

To establish the sandwich ELISA method, all the mAbs and HRP labeled mAbs were used as the capture and detection antibodies respectively in the pair-wise interaction analysis. Heat-killed *Pantoea stewartii subsp.stewartii* in 0.01 M PBS at the concentration of 1 × 10^8 cfu/mL and blank (0.01 M PBS) were tested respectively by the sandwich ELISA method. The combination which provided the highest positive/negative value (P/N value, the ratio of the optical density values of the positive test sample to negative sample) was selected as the pair for sandwich ELISA method.

1.4. Characterization of the Sandwich ELISA Method

A series of bacterial standards (1 × 10^9, 3.3 × 10^8, 1 × 10^8, 3.3 × 10^7, 1 × 10^7, 3.3 × 10^6, 1 × 10^6, 3.3 × 10^5, 1 × 10^5, 3.3 × 10^4 and 1 × 10^4 cfu/mL in 0.01 M PBS were tested by the sandwich ELISA method. The standard curve was generated with P/N value as the ordinate and concentrations of microorganism standards as the abscissa.

1.5. Cross-Reactivity of the Sandwich ELISA Method

Pantoea stewartii subsp.stewartii NCPPB 449 and other four plant pathogens: *B. glumae* NCPPB 3591, *X. oryzae pv. oryzicola* NCPPB 1150, *P. syringae pv. syringae* NCPPB 2844, and *X. oryzae pv. oryzae* NCPPB 3002 were tested by the sandwich ELISA method at a concentration of 10^8 cfu/mL.

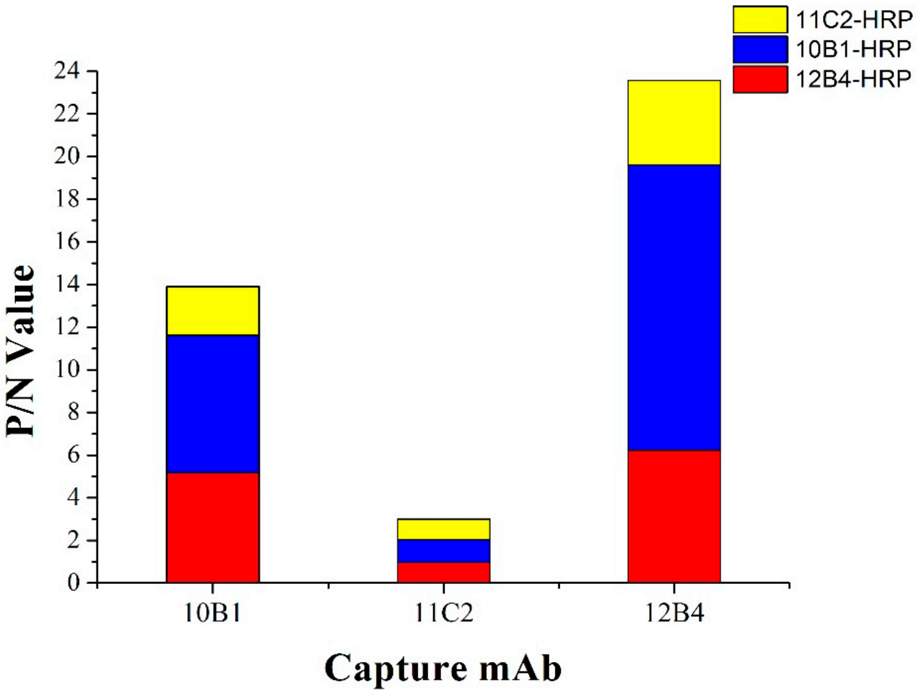

Figure S1. The pair-wise interaction analysis by sandwich ELISA (P/N value); Note: P/N value, the positive/negative value, which was the ratio of optical density value of the testing sample to the negative control sample.

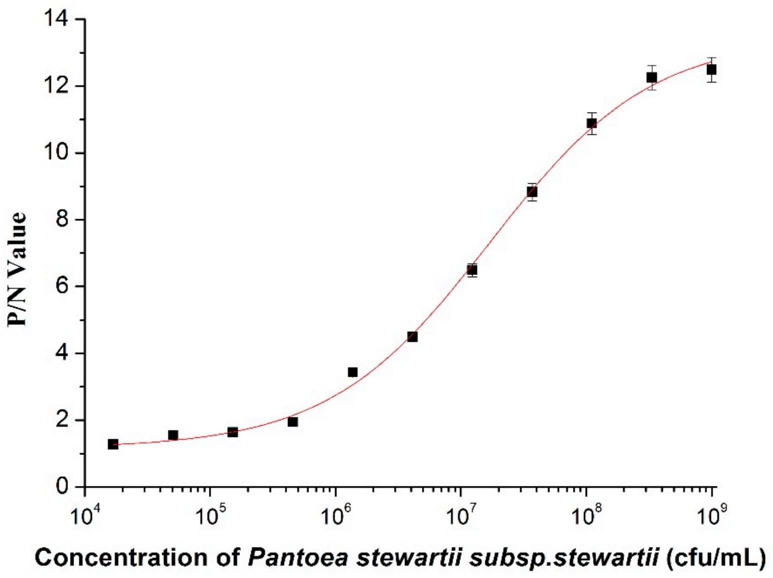

Figure S2. The standard curve of *Pantoea stewartii subsp.stewartii* in monoclonal sandwich ELISA; Note: P/N value, the positive/negative value, which was the ratio of optical density value of the testing sample to the negative control sample.

Table S1. The cross-reactivity of the sandwich ELISA method (n = 8).

Microorganism	Sandwich ELISA	
	OD_{450} Value	P/N Value
Pantoea stewartii subsp.stewartii NCPPB 449	1.901 ± 0.11	13.45 (+)
B. glumae NCPPB 3591	0.184 ± 0.017	1.54 (+)
X. oryzae pv. oryzicola NCPPB 1150	0.212 ± 0.011	1.72 (+)
P. syringae pv. syringae NCPPB 2844	0.240 ± 0.013	1.99 (−)
X. oryzae pv. oryzae NCPPB 3002	0.193 ± 0.012	1.30 (−)

Note: P/N value, the positive/negative value, which was the ratio of optical density value of the testing sample to the negative control sample. Values were calculated according to the formula P/N > 2.1. (+) means positive; (−) means negative.NCPPB: National Collection of Plant Pathogenic Bacteria, Harpenden, UK.

© 2015 by the authors; licensee MDPI, Basel, Switzerland. This article is an open access article distributed under the terms and conditions of the Creative Commons Attribution license (http://creativecommons.org/licenses/by/4.0/).
